# Micro-Structure of Hot Mix Asphalt Containing the 35/50 Bitumen Viewed in Terms of Excess Moisture in the Underlying Course of Pavement

**DOI:** 10.3390/ma13102230

**Published:** 2020-05-13

**Authors:** Paweł Mieczkowski, Bartosz Budziński, Robert Jurczak

**Affiliations:** Department of Civil Engineering, West Pomeranian University of Technology Szczecin, 70-310 Szczecin, Poland; Pawel.Mieczkowski@zut.edu.pl (P.M.); Robert.Jurczak@zut.edu.pl (R.J.)

**Keywords:** hot mix asphalt (HMA), compaction, air void content

## Abstract

Compaction of Hot Mix Asphalt (HMA) is a process aimed at obtaining the desired performance properties. Attainment of the required compaction can be hampered by external factors, which includes the presence of water. Water is known to cause quick lowering of the HMA temperature. The bottom face of the asphalt layers of a pavement is a sensitive point from the fatigue life point of view. In the site conditions, it is often difficult to obtain the required air void content at the bottom of an asphalt layer and excessive moisture content in the base course lying beneath the asphalt layer can be one of the causes. This article presents the results of tests carried out on a test section on which HMA was placed on an unbound aggregate base layer of varying moisture content. The material used for the binder course was asphalt concrete mixture composed of aggregate of minus 16 mm grading and 35/50 bitumen. Being relatively hard it is the most often specified bitumen for binder courses and also base courses. One of its characteristics is a considerable increase of viscosity with decreasing temperature, which hampers the process of compaction. The bulk specific gravity was measured to determine the variations in the air void content through the specimens. The complex modulus of elasticity and fatigue life were the other parameters which were determined on the specimens with different air void contents. The test results show worsening of the properties which have a decisive bearing on the service life of pavement.

## 1. Introduction

The Hot Mix Asphalt (HMA) micro-structure and performance characteristics are determined during the compaction process. The effectiveness of this process depends on the applied compaction effort, which is closely related to the mixture temperature [1,2,3]. The compaction effort is defined by the number of passes and speed of travel of the employed compacting roller on one path and on the compaction technique (defined by type of roller). The second parameter, namely temperature, depends on the rate of heat transfer inside the HMA layer and the dissipation of heat to the outside environment [4]. One of the environmental factors facilitating dissipation of heat from the mixture is the presence of water and this is owing to the properties of this substance, which include high specific heat (in the order of 4.186 kJ/kg·K) and the heat of vaporization (2257 kJ/kg at 100 °C) [5]. Water, which comes from precipitation or from wetting the steel drums of compacting rollers, can be present as moisture or even pools, present inside and/or on the surface of the underlying layer. Precipitation can also occur during the process of HMA placement. If it is only a light rain the contractor often takes a risk and, ignoring any prohibitions, decides to place the already delivered HMA anyway. After a direct contact of HMA with water occurs, the viscosity of the bitumen increases abruptly and reduces the temperature of the layer [6,7]. This is particularly harmful in the case of hard, i.e., low penetration binders with high softening point. This group of hard bitumens includes the 35/50 bitumen, commonly used in Poland for production of the asphalt mixtures used for binder course and road base layers for roads of KR3 to KR7 traffic service levels (according to the Polish classification system [8]). Rapid cooling of bituminous mixtures containing a binder of this kind causes problem in the process of compaction, so big that in many cases it is impracticable to achieve the required density. The process of compaction of HMA can be affected by the presence of even a small amount of water with consequences that are not always evident from the compaction index determined on the pavement cores. This can result in an increased air void content and a consequential decrease of the HMA’s resistance to the environmental factors [9]. As a consequence, premature distress, including potholes, cracking and spalling, can occur on the top surface of the pavement [10,11]. The cause of this distress is the accelerated aging resulting in the deterioration, namely hardening of the binder due to the access of atmospheric oxygen and UV [12,13]. If the underlying layer contains excess moisture, then a higher air void content at the bottom of the asphalt layers will affect the fatigue life of pavement and lead to premature initiation of cracks at the bottom of the asphalt layers due to tensile strains [14]. Moreover, a loss of bond between the pavement courses can occur if precipitation occurs during placement of HMA [15,16]. The presence of water on the top of an underlying asphalt layer (for example road base) can result in a loss of bond between the asphalt layers (for example between the road base and the binder course). This kind of defect can also be caused by spraying of asphalt emulsion on the surface of the underlying layer just in front of the asphalt spreader [17]. After break-up of the bituminous emulsion water can no longer evaporate or drain from the surface and acts as a kind of bond breaker between the asphalt layers of pavement. Thus, a lack of bond between the pavement layers ensues, considerably decreasing the fatigue life of the pavement [18]. The problem of subbase unbound is important among others due to the unpredictability of weather conditions and the possibility of conducting construction works during or shortly after rain stops. The authors considered the problem of subbase unbound on a technical scale by performing tests on materials taken from experimental sections which allowed to determine the parameters of laboratory samples. Based on the results obtained on the experimental sections the impact of the work of compaction and subbase unbound of void content and the density of asphalt layers was determined. Laboratory tests covered a range of HMA parameters, the results of which allowed determining the effect of subbase unbound on the main HMA parameters.

## 2. Test Section

### 2.1. Materials and Testing Program

#### 2.1.1. Properties of the Bitumen

The bitumens used for the production of paving mixtures include moderately hard bitumens of 50/70 grade, harder bitumens of 35/50 grade and binders from a quite large group of polymer modified bitumens, for example PMB 25/55–60) [19]. However, for the cost reasons and to ensure the desired deformation resistance of the pavements placed on heavily trafficked roads (traffic service levels KR3 to KR7 [8]) the 35/50 bitumen is the material of choice for production of the base and binder course mixes. This being so, this bitumen type was used for the production of the Asphalt Concrete (AC 16 W 35/50) binder course mixture used to construct the test section pavement. The essential classification parameters of this binder are given in the Table 1 below.

The penetration index I_p_ values were found as in Equation (1) by determining the bitumen temperature susceptibility taking into account its 25 °C penetration grade (P) and softening point (T_R&B_) and utilizing the formula according to EN 12591 [24]:

(1) $$I_{p}=\frac{20\times T_{R\&B}+500\times \mathrm{lgP}-1952}{T_{R\&B}\times \mathrm{lgP}+120}$$

The temperature range of plasticity (plasticity range, PR) of the binder, dependent on its softening point (T_R&B_) and breaking point (T_Fraass_), was determined by Equation (2) in compliance with the requirements laid down in PN-EN 14023:2011/Ap:2020-02 (National Annex NA) [25] according to the following formula:

(2) $$\mathrm{PR}=T_{R\&B}-T_{\mathrm{Fraass}}$$

**Figure 1:**
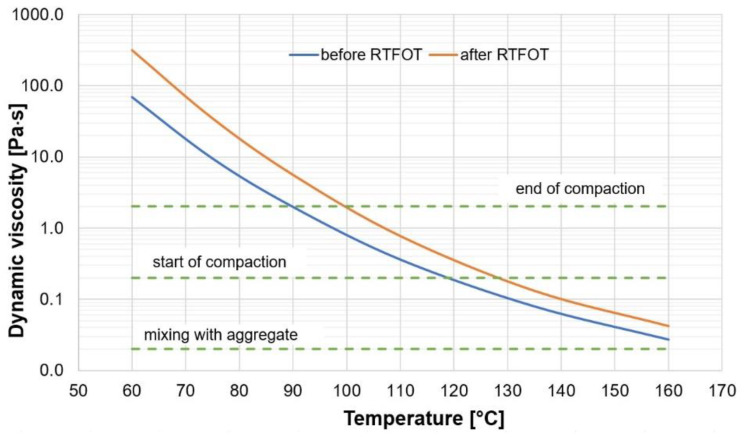
Viscosity of the 35/50 bitumen vs. temperature (before and after RTFOT aging).

Additionally, the dynamic viscosity of the 35/50 bitumen was measured before and after RTFOT aging according to EN 13302 [26] at the test temperatures of 60, 90, 135, and 160 °C. The test data are displayed in Figure 1.

The above values of the dynamic viscosity of bitumen show that asphalt mixtures containing the 35/50 bitumen required relative high temperatures both during production (over 160 °C) and during compaction (100–130 °C). The presence of water, acting as an additional cooling factor can considerably shorten the compaction time, thus increasing the in-situ air void content.

#### 2.1.2. Properties of the Asphalt Used on the Test Section

**Figure 2:**
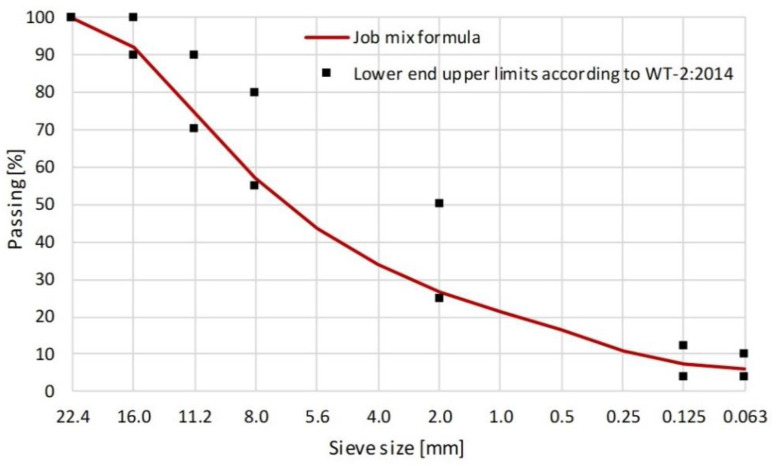
Grading of the mineral mixture in the AC 16 W 35/50 asphalt concrete.

For the purposes of this research a test section was constructed to assess the effect of water on the micro-structure of the compacted HMA layer. The experimental verification concerned the effect of excess moisture in the underlying layer, the compaction effort and the thickness of HMA layer on the values of bulk specific gravity across the layer. The pavement was made of asphalt concrete type mixture containing 35/50 bitumen, designated for binder course construction. The aggregate grading parameters are displayed in Figure 2 below. For the mixture design and main properties see Table 2 and Table 3. The asphalt concrete mixture was specified in compliance with the Polish design manual No. WT-2:2014 [27].

**Figure 3:**
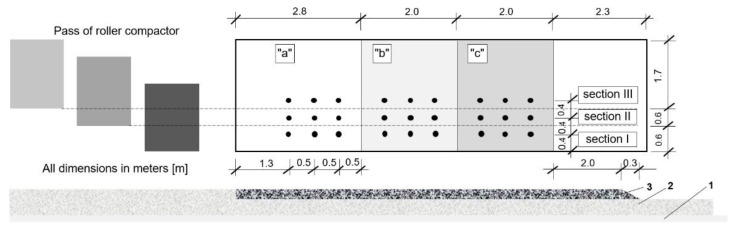
Schematic representation of the test section showing the effect of the number of roller passes and the content of moisture in the underlying layer on the relative compaction of the asphalt mixture (AM) layer. Legend: 1–C_3/4_ cement-bound layer; 2–layer of # 0/31.5 mm mechanically compacted crushed aggregate; 3–AC 16 W binder course, 8 and 4 cm in thickness; zones of different moisture contents: 2% (**a**), 6−8% (**b**), and 15−18% (**c**) respectively; sections varying in terms of the applied compaction effort (compactor roller was employed for the work): I—two static passes, II—six passes including two vibratory ones, III—10 passes including 4 vibratory ones.

The AC 16 W 35/50 binder course mixture was placed on a suitably prepared underlying course, which in this case was # 0/31.5 mm crushed aggregate base of varying moisture content, as schematically shown in Figure 3. Just before HMA placement the essential parameters of the underlying layer were checked, including flatness, bearing capacity, compaction and moisture content. The area was divided into three test zones differing in terms of the moisture content in the top part of the layer, i.e., from the top to 6 cm below the surface. In the first test zone (designated “a”) natural moisture content of 2% was not changed and in the other zones water was poured to increase the moisture content to 6−8% in the test zone “b” and 15−18% in the test zone “c”. The moisture content was measured with a pin-style moisture meter utilizing resistive moisture measuring technique. The JT T-90 MM type moisture meter has been used. The laboratory tests showed that the differences between the device readings (with the mean of 5 readings) and moisture tests results in accordance to EN 1097-5 did not exceed 1%. About 10 readings have been made for each area. On the base, prepared as described above the tested HMA mixture was placed by an asphalt spreader. The paver travelled at a speed of 2 m/min. The initial temperature of HMA was ca. 155 °C. During the work the ambient air temperature was 18 °C at ca. 70% Relative humidity (RH). The mixture was placed on a 9.1 m long by 2.7 m wide surface. The process of compaction was commenced after about 3 min. from placement. A nine ton vibratory roller (type CC322) was employed, travelling at a speed of 3 km/h in 1 min work cycles. The first two passes from the edge of the layer, in the forward and reverse directions of travel, were static. After that the roller was shifted to 60 cm from the edge and the next passes were done as follows: the first pass, in the forward direction–static, the second and third passes, in the forward and reverse directions–with vibration and the fourth pass, in the reverse direction–static. This procedure was repeated after the next shift, this time by 40 cm. In this way, each section was compacted as follows (Figure 3):

- section I—two static passes,
- section II—four static passes plus two vibratory passes,
- section III—six static passes plus four vibratory passes,

The total duration of the HMA compaction process was 10 min. The compacted thickness of the HMA layer was 8.0 ± 0.2 cm.

Once the placed asphalt mixture had cooled down, three samples (100 mm cores) were taken from each zone and from each test section, giving twenty seven samples in total. Next the 8 cm thick binder course was removed and replaced with a 4 cm thick binder course. The weather conditions and the moisture content in the base were much the same as during the previous placement.

### 2.2. Results and Discussion

**Figure 4:**
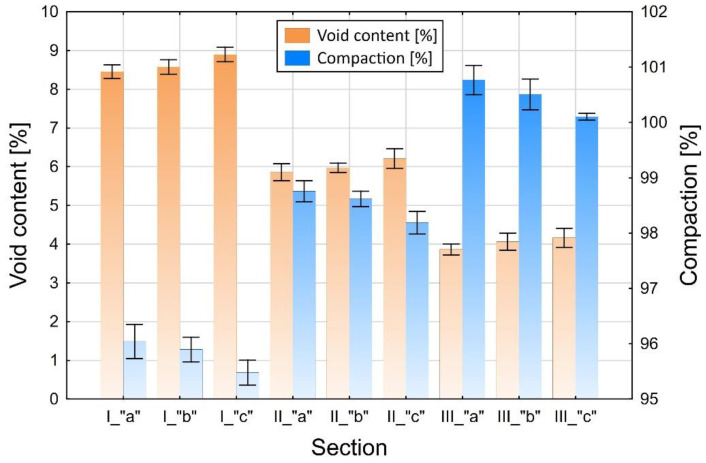
Compaction and air void content in the cores taken from the 8 cm thick binder course depending on the location. The zone and section designations are the same as in Figure 3.

**Figure 5:**
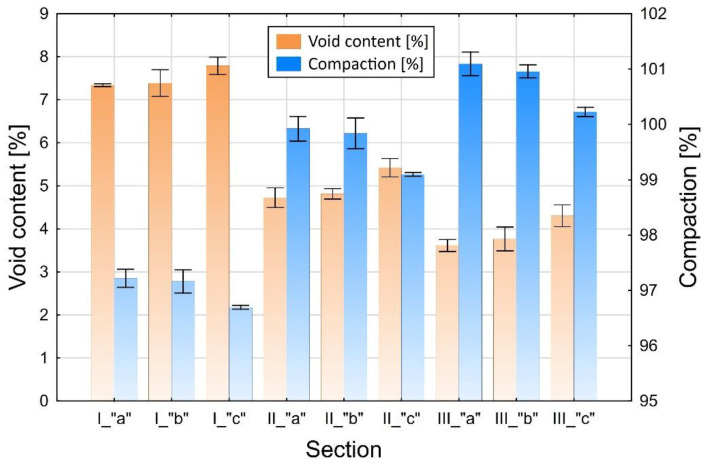
Compaction and air void content in the cores taken from the 4 cm thick binder course depending on the location. The zone and section designations are the same as in Figure 3.

The samples of asphalt concrete were cut from the test section (Φ = 100 mm cores), as shown in Figure 3. The bulk specific gravity was determined on the entire cores using method B [28] as per EN 12697-6. The in-place air void content and compaction values were determined for the complete layer according to EN 12697-8 (Section 4) [29]. The results, each calculated as an average of three cores for the respective layers made of AC 16 W mixture (8 cm and 4 cm thick) are presented in Figure 4 and Figure 5 for different compaction efforts and moisture contents in the respective sections and zones.

**Figure 6:**
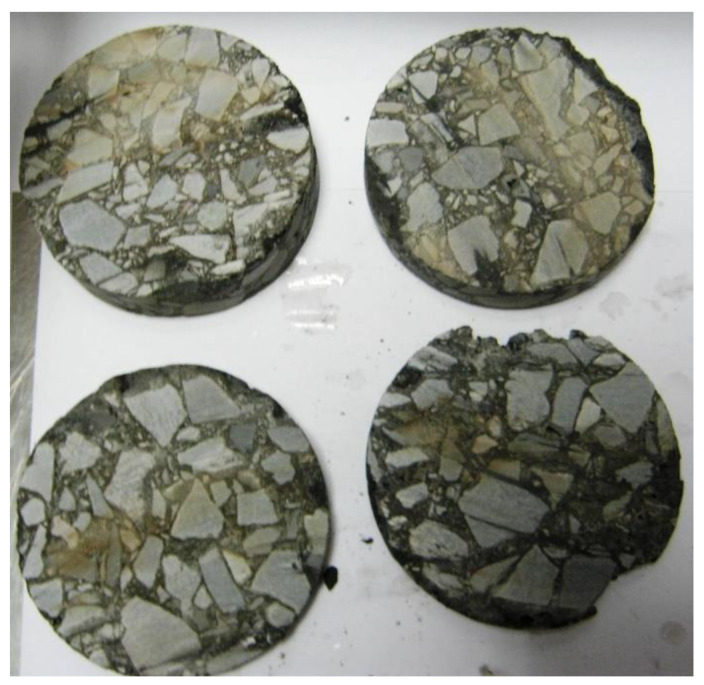
Sample taken from the 4 cm thick binder course, sliced into 0.8–1.0 cm slices.

**Figure 7:**
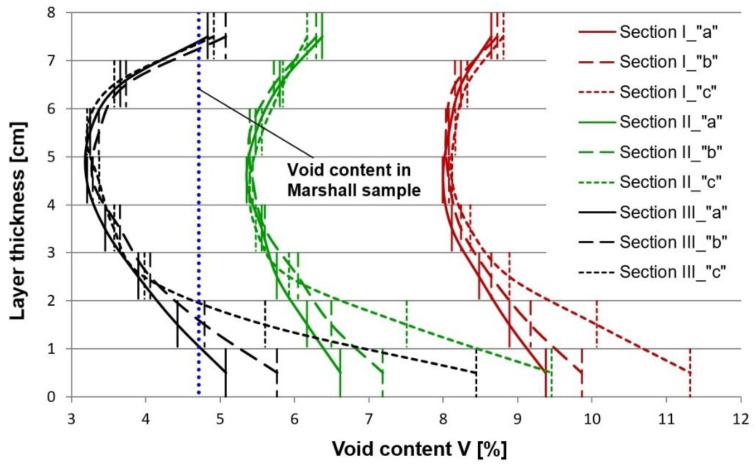
Air void content (V_m_) in the 8 cm thick binder course, determined on the sliced specimens, depending on the number of passes of the compactor roller. The designations of the sections (I, II, and III) and the test zones of different moisture content (a, b, and c) are the same as used in Figure 3.

**Figure 8:**
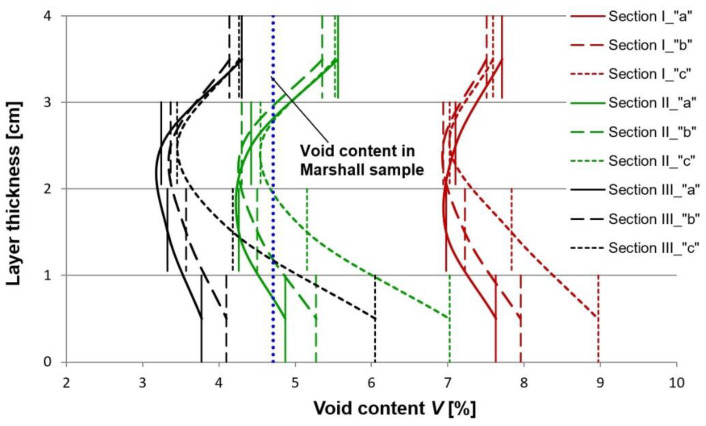
Air void content (Vm) in the 4 cm thick binder course, determined on the sliced specimens, depending on the number of passes of the compactor roller. The designations of the sections (I, II and III) and the test zones of different moisture content (a, b, and c) are the same as used in Figure 3.

In the next step the samples were sliced into ca. 0.8–1.0 cm thick slices (Figure 6). Next the bulk specific gravity was determined on these slices according to method C, as per EN 12697-6 (sealed specimen). The average air void content values, taking account of the location inside the layer are presented in Figure 7 and Figure 8.

**Figure 9:**
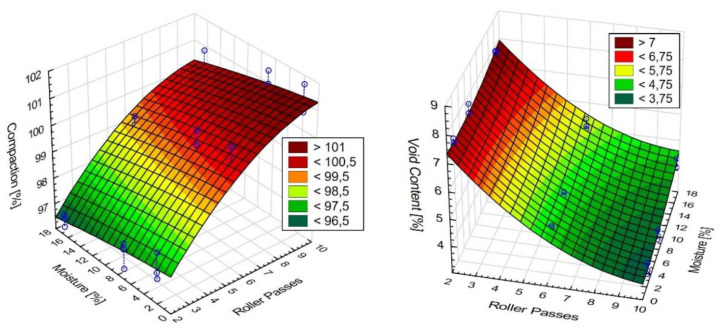
The influence of free space content and compaction on the number of roller passes and soil moisture.

The observed variation of the test results obtained for the respective the sections, differing in terms of the number of passes and the test zones (a, b, and c) indicates the high degree of complexity of the studied problem. We can conclude, on the basis of these results, that the compaction effort is the factor which has a decisive bearing on the obtained micro-structure of asphalt. The increase of the air void content in the bottom part of the sample was expected and is consistent with the results of other reported studies [30,31]. However, it is important note, that the considerable increase of the air void content in the bottom part of the specimen (within 1–2 cm from the bottom face) results from the excess moisture in the base, which should be considered an undesired effect. The higher content of free space in the upper part of layer against its center springs from faster cooling of this part and increase of asphalt tenacity. Faster temperature drops in the upper part of layer (about 1.0–1.5 cm) results from the heat flow. In the middle of the layer the heat flow takes place by conduction whereas on the surface and in its upper part by convection (forced and free) also as a result of the impact of water used to sprinkle the steel roller drum. Heat consumption as a result of convection and water is much higher than for conduction, thus rapid and significant temperature drops are set down. The influence of free space content and compaction on the number of roller passes and soil moisture is shown in Figure 9. On the construction site, the main importance in achieving the required compaction and free space content for the entire thickness of the layer is the number of roller passes and then later humidity.

## 3. Laboratory Testing

### 3.1. Materials and Test Procedures

**Figure 10:**
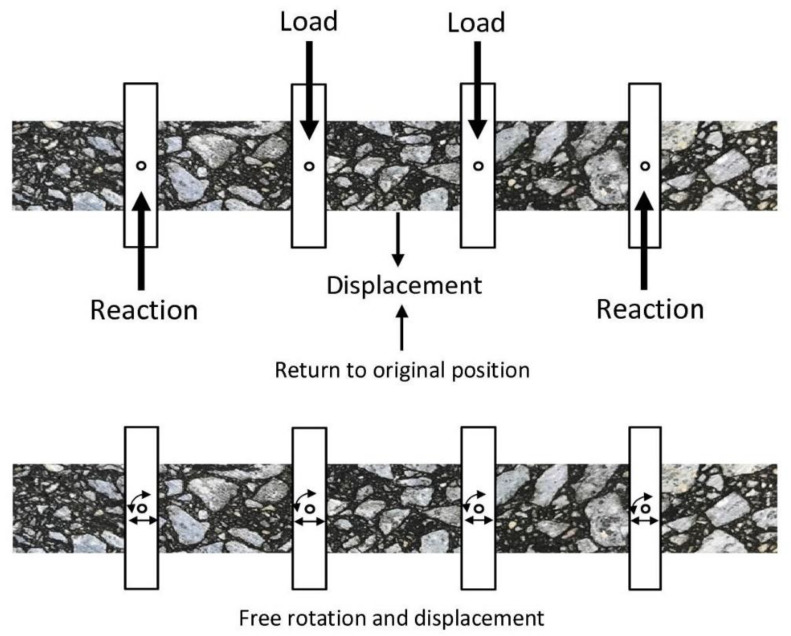
Schematic representation of the asphalt concrete prismatic beam specimens clamped on the test machine.

The results obtained from the testing the asphalt concrete samples taken from the test section show that excess moisture in the underlying layer increases the air void content in the bottom part of the overlying asphalt layer. It was there much higher than in in the central area and also in the sub-surface portion of the layer. Therefore, it was decided to carry out laboratory testing of lab-prepared specimens as the next stage of this research. These specimens were made of AC 16 W 35/50 binder course mixture, the same as used on the test section of pavement. The specimens were compacted to 96% (IVC—increased air void content) and 100% (MVC—Marshall air void content) in relation to the design value—see the job mix formula in Table 3. The specimens were cut from the lab-prepared asphalt slabs, compacted with plate compactor to obtain the two pre-defined air-void contents, i.e., Vm = 5.1% (MVC—100% relative compaction) and Vm = 8.8% (IVC—96% relative compaction). The respective determinations were analyzed together. In this way, the results covered a wider range of air void content values. An increased air void content can affect the complex modulus (E*) and the fatigue life (log_10_N) of the asphalt concrete and decrease, as a result, the load bearing performance and service life of the entire pavement. The tests to determine the values of the above-mentioned parameters were carried out on two series of specimens differing in terms of the air void content, resulting from different compaction. The four-point bending test on prismatic specimens (4PB-PR) was employed. The test set-up is schematically represented in Figure 10.

The complex modulus E* was determined at the temperature of 10 °C and 10 Hz frequency as per EN 12697-26 [32]. It was determined in the 100th load cycle at 50 μm/m strain amplitude. The test was carried out on two series of specimens (6 No. each) differing in terms of the air void content. The air void content obtained for the specimens in the first series approximated the value obtained in the Marshall test, i.e., 4.5–5.6%. In the second series the air void content fell in the range of 7.9 to 9.4%. The fatigue life test was carried out at the temperature of 10 °C and 10 Hz frequency at the constant strain amplitudes of 90, 110, and 130 μm/m, as per EN 12697-24 [33]. The fatigue life (log_10_N—number of load cycles) was determined for the cycles during which the stiffness modulus value decreased to 50% of the initial value, which was determined during the 100th cycle. The tests were carried out on two series of specimens, six for each strain amplitude, varying in terms of the air void content. In the first series, the air void content fell in the range of 4.4 to 5.6%. In the second series the air void content fell in the range of 7.9 to 9.5%.

### 3.2. Results and Discussion

**Figure 11:**
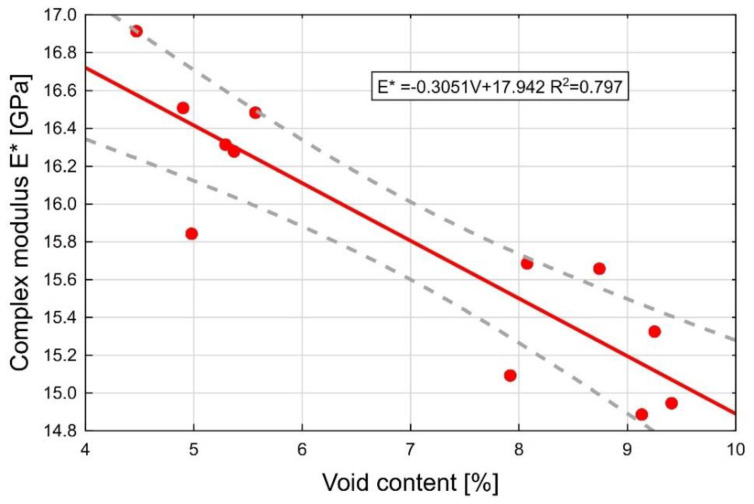
The effect of the air void content in AC 16 W 35/50 asphalt concrete on the complex modulus of the layer.

According to the test result there is a strong correlation between the values of the air void content and the complex modulus (Figure 11) This is confirmed by the R-squared value of R^2^ = 0.797 and also by the curve of best fit.

**Figure 12:**
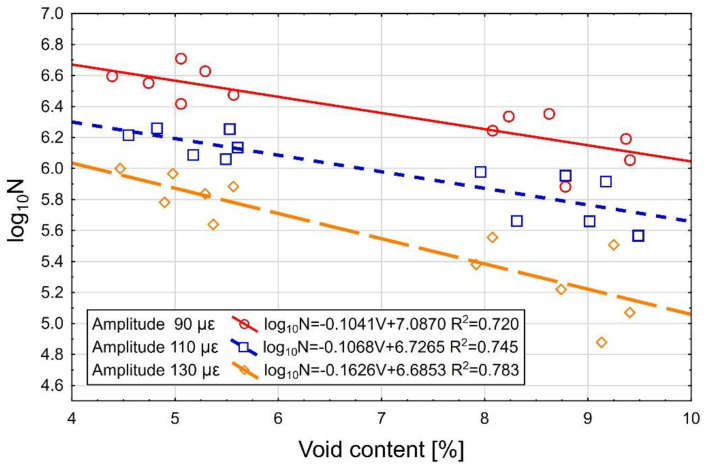
The effect of the air void content in AC 16 W 35/50 asphalt concrete on the fatigue life of the layer (log_10_N) for the strain amplitudes of 130, 110 and 90 μm/m.

The effect of the air void content on the fatigue life is analyzed below. The log_10_N fatigue life test results depending on the air void content are displayed in Figure 12 below.

When the strain amplitude is taken into account, the analysis reveals correlation between the air void content and the fatigue life of the specimens. This is confirmed by the R-squared values falling in the range of R^2^ = 0.720–0.783 (with the exact value depending on the strain amplitude) and also by the curve of best fit (Figure 12). Attention is drawn to the inclination of the fatigue line in relation to the horizontal axis for the respective strain amplitudes expressed by the slope factor. The greatest inclination (and thus the variations of log_10_N fatigue life) are observed for the 130 μm/m strain amplitude. This means that the effect of the air void content on the fatigue life increases with the increase of the strain amplitude.

**Figure 13:**
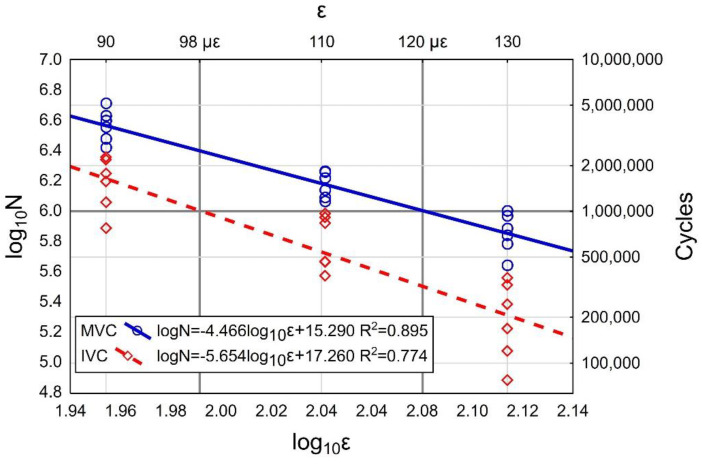
Visual representation of the fatigue behavior of the AC 16 W 35/50 mixtures determined on the two series of specimens (*MVC*—Marshall air void content, *IVC*—increased air void content).

The effect of the air void content on the parameters of the AC 16 W 35/50 asphalt concrete is the most evident in the fatigue curve. The graph in Figure 13 represents the relationship between the log_10_N fatigue life and the strain amplitudes for the specimens of different air void contents. Also in this case the inclination angle of the trend line in relation to the horizontal axis is greater for the specimens with a higher air void content (IVC). The values of the limit strain ε, which is the strain at which the complex modules decreased by 50% after one million cycles, differ by appreciable amounts. In the case of properly compacted specimens (MVC) the limit strain was 120 μm/m while for specimens with an increased air void content (IVC) this value approximated 98 μm/m. This indicates a very strong influence of the air void content on the fatigue life of asphalt mixtures, in particular when they are used for the bottom layers of the pavement structure.

The least squares method was employed to derive an equation describing the relationship between the number of cycles, the strain amplitude and the air void content. The equation has been written in the following general form Equation (3):

(3) $$\mathrm{Cycle}=10^{{\;b}_{1}+b_{2}\mathrm{amplitude}+b_{3}V}$$

The results of the analysis are given in Table 4 below.

The value of *p* = 0.0000 confirms the significance of all the coefficients. Finally, we obtain the function describing the relationship between the number of cycles, the strain amplitude and air void content expressed by the following Equation (4):

(4) $$\mathrm{Cycle}=10^{\;8.725-0.0185\mathrm{amplitude}-0.0972V}$$

**Figure 14:**
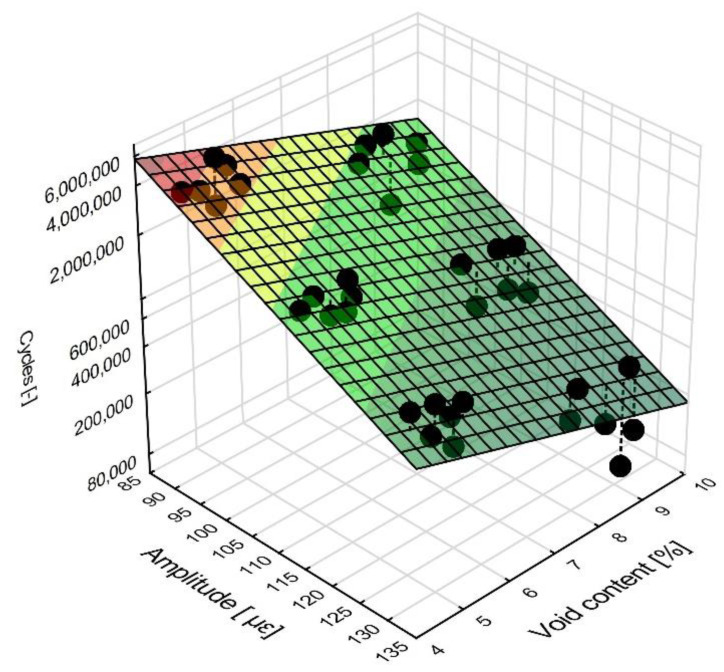
The relationship between the number of cycles, the strain amplitude, and the air void content showing the locations of the measurement points.

This relationship is shown in the graph in Figure 14 below. The validity of the Equation (4) was confirmed by the analysis of residuals and the analysis of the graph.

## 4. Final Conclusions

1. The 35/50 bitumen is classified in the group of relatively hard binders requiring high application temperatures. A rapid increase of the viscosity of bitumen resulting from contact with water can considerably hamper the process of compaction of mixtures that contain this bitumen.
2. The results of the tests carried out on the entire cores show that the amount of effort applied by the compactor and the time frame of the process have a decisive bearing on obtaining the desired compaction. The number of passes should be specific to the type of mixture, its temperature and, last but foremost, the compacted layer thickness. The excessive amount of effort leads to over-compaction, especially in the center of the layer, which in some cases can decrease its resistance to permanent deformations.
3. An insufficient number of passes, in turn, will result in an increased air void content through the entire thickness of the layer, the most in the subsurface portion, resulting in a decreased fatigue life or weather resistance in the case of the wearing course. The example of the lack of density can be section I in which the samples had a free space above 7% in the entire cross-section (with a layer thickness 4 and 8 cm).
4. While the excess moisture in the underlying layer plays some role, the variation of the air void content in the asphalt layer in the areas of the highest moisture content (zones “a” and “c”) determined on the entire specimens (prior to slicing) are small, i.e., maximum 1%. The variation of the results and the actual distribution of pores in the mixture became apparent in the sliced specimens. It is evident that high amounts of moisture inhibit obtaining the desired air void content in the mixture, in particular at the bottom of the layer. An increased porosity was noted within 1–2 cm from the bottom face of the layer where the air-void content reaching 7–12% was noted. This affects the parameters relevant to the fatigue performance of the mixture in the zone subjected to the highest tensile stress levels.
5. An increase of the air void content causes a decrease of the value of complex modulus E*. In researches with the free space increase by of 3.7% average, the decrease of complex module was almost 7%. As a consequence, higher tensile strains due to traffic loading can be expected at the bottom of the pavement structure due to a smaller stress distribution area.
6. An increased air void content in the mixture considerably reduces the value of the limit strain ε. A decrease by 22 μm/m from 120 to 98 μm/m was noted for the analyzed mixture.
7. An increase of the tensile strains at the bottom of inadequately compacted asphalt layers in combination with impaired fatigue performance parameters will reduce the fatigue life of the entire road pavement. This will cause premature fatigue cracking, initiated at the bottom of asphalt layers.
8. An increased air void content at the bottom of an asphalt layer caused by the presence of moisture in the underlying layer will considerably decrease the fatigue life of the entire pavement. This effect will be noticeable even if the layer as a whole simultaneously satisfies both the applied criteria, i.e., degree of compaction and the air void content.

## Figures and Tables

**Table 1:** The values of the main parameters applied for classification of 35/50 bitumen before and after Rolling Thin Film Oven Test (RTFOT as per EN 12607-1 [20]).

Type of Test	Standard	Test Value	
		Before RTFOT	After RTFOT
Penetration (P) (25 °C, 100 g, 5 s), [× 0.1 mm]	EN 1426:2015-08 [21]	46.2 ± 1.1	46.2 ± 0.6
Softening point (T_R&B_) (5 °C/min), [°C]	EN 1427:2015-08 [22]	55.9 ± 0.3	59.2 ± 0.4
Fraass breaking point (T_Fraass_), [°C]	EN 12593:2015-08 [23]	−11.9 ± 1.7	−9.4 ± 0.9
Penetration index (I_p_), [−]	EN 12591:2010 (Annex A) [24]	−0.018	−0.259
Plasticity range (PR), [°C]	PN-EN 14023:2011/Ap2:2020-02, National Annex NA [25]	67.8	68.6

**Table 2:** Components of the AC 16 W 35/50 binder course mixture.

Materials	Bulk Specific Gravity [g/cm^3^]	Composition of	
		Mineral Mixture (MM)	Asphalt Mixture (AM)
Coarse agg. # 11/16 (granodiorite)	2.727	24.0	22.9
Coarse agg. # 8/11 (granodiorite)	2.751	20.0	19.1
Coarse agg. # 5/8 (granodiorite)	2.745	16.0	15.3
Coarse agg. # 2/5 (granodiorite)	2.745	12.0	11.4
Crushed fine agg. # 0/2 (granodiorite)	2.745	24.0	22.9
Filler agg. (limestone)	2.747	4.0	3.8
Bitumen 35/50	1.030	–	4.6
Adhesive additives (by weight of binder)	–	–	0.3

**Table 3:** Properties of the AC 16 W 35/50 mixture.

Property		Requirements According to WT-2 2014	Value
Maximum density of HMA, EN 12697-5, method A in water [Mg/m^3^]		–	2.511
Bulk specific gravity of HMA EN 12697-6, method B [Mg/m^3^]		–	2.419
Air Void Content V, EN 12697-8 [%]		4.0–7.0	5.17
Rutting resistance, EN 12697-22, 60 °C, 10,000 cycles	WTS_AIR_ [mm/10^3^ cycles]	≤0.10	0.06
	PRD_AIR_ [%]	≤5.0	4.7
Stiffness, EN 12697-26, 4PB-PR, 10 °C, 10 Hz [MPa]		–	16,290
Resistance to fatigue, EN 12697-24, 4PB-PR, 10 °C, 10 Hz, *ε* = 115 μm [10^6^ cycles]		–	1.23

**Table 4:** The results of the regression analysis.

Coefficient	Result	*p*
b_1_	8.725	0.0000
b_2_	−0.0185	0.0000
b_3_	−0.0972	0.0000
